# Supplementary material for: Metabolomic Biomarkers of Dietary Approaches to Stop Hypertension (DASH) Dietary Patterns in Pregnant Women
Source: Nutrients. 2024 Feb 8;16(4):492. doi: 10.3390/nu16040492 (PMC10892314; doi:10.3390/nu16040492)
Supplement: Supplementary file 1 [file nutrients-16-00492-s001.zip › nutrients-2810636-supplementary.pdf]

Supplement Materials

Supplementary Table S1. Associations of 92 individual fasting plasma metabolites with DASH score in linear regressions, the Fetal Growth Studies–Singleton Cohort (FGS).

Superclass	Class/Subclass	Metabolites	Coefficient	*P*-values[1]	Adjusted *P*-values[2]	Food group
Benzenoids	Benzoic acids	Benzoic acid	0.11	0.008	0.04	Low fat dairy
Homogeneous non-metal compounds	Homogeneous other non-metal compounds	Hydroxylamine	0.27	<0.001	0.01	
Organic acids and derivatives	Amino acids	Asparagine	0.13	0.005	0.03	Sodium
		Beta-alanine	0.18	0.004	0.03	Low fat dairy
		Glutamic acid	-0.20	0.005	0.03	
		Glycine	-0.19	<0.001	0.003	Low fat dairy
		N-acetylornithine	0.28	0.004	0.03	
		Taurine	-0.49	0.002	0.02	
	Amino acid derivatives	Hydroxycarbamate	0.32	<0.001	<0.001	Low fat dairy
Organic oxygen compounds	Short-chain hydroxy acids/ Monosaccharides	2,3-dihydroxybutanoic acid	0.22	0.003	0.02	Sodium
	Carbohydrate/ Disaccharides	Maltose	-0.40	0.003	0.02	
	Carbohydrate/ Monosaccharides	Xylitol	0.16	0.001	0.01	
Organoheterocyclic compounds	Vitamin B3	Nicotinic acid	0.50	0.003	0.02	Fruit
	Pyrrolidines	Maleimide	0.25	0.006	0.04	
Lipids and lipids-like molecules	Prenol lipids/ Vitamin E	Gamma-tocopherol	-0.19	0.004	0.03	
	Fatty Acyls	Acylcarnitine C18:2	-0.17	0.008	0.04	
		Adipic acid	0.17	0.003	0.02	

	Dodecanoic acid	0.22	0.002	0.02	Fruit & Low fat dairy
Glycerolipids	DG (34:1)	0.22	0.004	0.03	Fruit & Low fat dairy
	DG (38:5)	0.18	0.005	0.03	
	TG (14:0/14:0/14:0)	0.61	0.002	0.02	Low fat dairy
	TG (44:1)	0.69	0.001	0.01	Low fat dairy
	TG (44:1)	0.61	<0.001	0.008	Low fat dairy
	TG (46:0)	0.67	<0.001	0.002	Low fat dairy
	TG (46:1)	0.66	<0.001	0.002	Low fat dairy
	TG (46:1)	0.55	<0.001	0.002	Low fat dairy
	TG (46:2)	0.55	0.003	0.02	Low fat dairy
	TG (46:2)	0.49	0.001	0.01	Low fat dairy
	TG (48:0)	0.49	<0.001	0.01	
	TG (48:0)	0.43	0.002	0.02	
	TG (48:1)	0.40	<0.001	0.003	Low fat dairy
	TG (48:1)	0.42	<0.001	0.001	Low fat dairy
	TG (48:2)	0.40	<0.001	0.004	Low fat dairy
	TG (48:2)	0.31	<0.001	0.002	Low fat dairy
	TG (48:3)	0.26	0.010	0.05	Low fat dairy
	TG (49:0)	0.47	<0.001	0.004	Low fat dairy
	TG (49:0)	0.40	<0.001	0.003	Low fat dairy
	TG (49:1)	0.42	<0.001	0.003	Low fat dairy
	TG (49:1)	0.38	<0.001	<0.001	Low fat dairy
	TG (49:2)	0.31	0.003	0.02	Low fat dairy & sodium
	TG (49:2)	0.29	<0.001	0.00	Low fat dairy

	TG (49:3)	0.23	0.007	0.04	Low fat dairy & sodium
	TG (50:0)	0.28	0.009	0.05	
	TG (50:1)	0.21	0.003	0.02	
	TG (50:1)	0.20	<0.001	0.00	
	TG (51:1)	0.31	0.004	0.03	Low fat dairy
	TG (52:2)	-0.08	0.001	0.01	
	TG (53:4)	-0.18	0.010	0.05	Red & processed meat
	TG (54:3)	-0.16	0.003	0.02	
	TG (54:3)	-0.15	<0.001	0.01	
	TG (54:6)	-0.27	0.006	0.04	
	TG (56:1)	0.36	0.003	0.02	Low fat dairy
	TG (56:1)	0.36	0.002	0.02	Low fat dairy
	TG (58:1)	0.38	0.004	0.03	Low fat dairy
	TG (58:1)	0.81	<0.001	<0.001	Low fat dairy
	TG (58:2)	0.29	0.005	0.03	Low fat dairy
	TG (58:8)	0.31	0.009	0.05	
	TG (60:2)	0.31	0.007	0.04	Fruit & low fat dairy
	TG (60:2)	0.25	0.007	0.04	Low fat dairy
Glycerophospholipids	LPC (14:0)	0.22	0.007	0.04	Low fat dairy; & sodium
	PC (28:0)	0.53	<0.001	0.002	Low fat dairy
	PC (30:0)	0.38	<0.001	<0.001	Low fat dairy & sodium
	PC (30:1)	0.42	<0.001	0.004	Low fat dairy & sodium

PC (31:0)	0.25	<0.001	0.002	Low fat dairy & whole grain & sodium
PC (32:1)	0.33	<0.001	0.001	Low fat dairy
PC (32:3)	0.34	<0.001	0.001	Low fat dairy
PC (33:0)	0.23	<0.001	0.004	Low fat dairy & sodium
PC (33:1)	0.25	<0.001	0.001	Low fat dairy & whole grain & sodium
PC (34:1)	0.11	<0.001	0.00	Low fat dairy & sodium
PC (34:4)	0.25	0.002	0.02	Low fat dairy & sodium
PC (35:1)	0.19	<0.001	0.01	Low fat dairy & sodium
PC (35:3)	0.23	<0.001	0.00	Low fat dairy & sodium
PC (36:1)	0.15	<0.001	0.002	Low fat dairy & sodium
PC (36:3) B	0.24	<0.001	0.01	Low fat dairy & sodium
PC (36:4) C	0.07	<0.001	0.001	Fruit
PC (36:5) B	0.41	<0.001	0.002	
PC (36:6)	0.35	<0.001	0.002	Low fat dairy & sodium
PC (37:3)	0.20	0.002	0.02	Low fat dairy & sodium & red and processed meat
PC (38:2)	0.20	<0.001	0.01	Low fat dairy & sodium

	PC (38:3)	0.23	<0.001	<0.001	Low fat dairy & sodium
	PC (38:4) B	0.16	<0.001	<0.001	Low fat dairy & sodium
	PC (38:5) A	0.15	<0.001	0.01	
	PC (42:5)	0.17	0.004	0.03	Low fat dairy & sodium
	PC (42:6)	0.60	<0.001	<0.001	Low fat dairy
Sphingolipids	SM (d30:1)	0.30	<0.001	0.004	Low fat dairy & sodium
	SM (d32:0)	0.29	<0.001	0.003	Low fat dairy & sodium
	SM (d32:1)	0.23	<0.001	0.01	Low fat dairy & sodium
	SM (d40:2)	0.17	0.006	0.04	Low fat dairy & sodium
	SM (d43:1)	0.35	0.010	0.05	Low fat dairy & sodium
Sterol lipids	CE (20:3)	0.58	<0.001	0.003	
	CE (20:5)	0.36	0.007	0.04	Fruit
	CE (20:5)	0.40	0.004	0.03	

[1] Linear regression models were adjusted for age (years), race (non-Hispanic White, non-Hispanic Black, Hispanic, Asian & Pacific Islander), education (high-school degree or less, associated degree, bachelor's degree or more), pre-pregnancy BMI (kg/m^2), physical activity (metabolic equivalent hours per week).
[2] Benjamini-Hochberg procedure was applied to adjust the multiple comparisons and statistically significance was considered for the false discovery rates (FDRs) <0.05.
Abbreviations: BMI, body mass index; CE, cholesteryl ester; DASH, Dietary Approaches to Stop Hypertension; DG, glycerolipids; FDR, false discovery rate; NICHD, National Institute of Child Health and Human Development; PC, phosphatidylcholine; SM, sphingomyelin; TG, triacylglycerol.

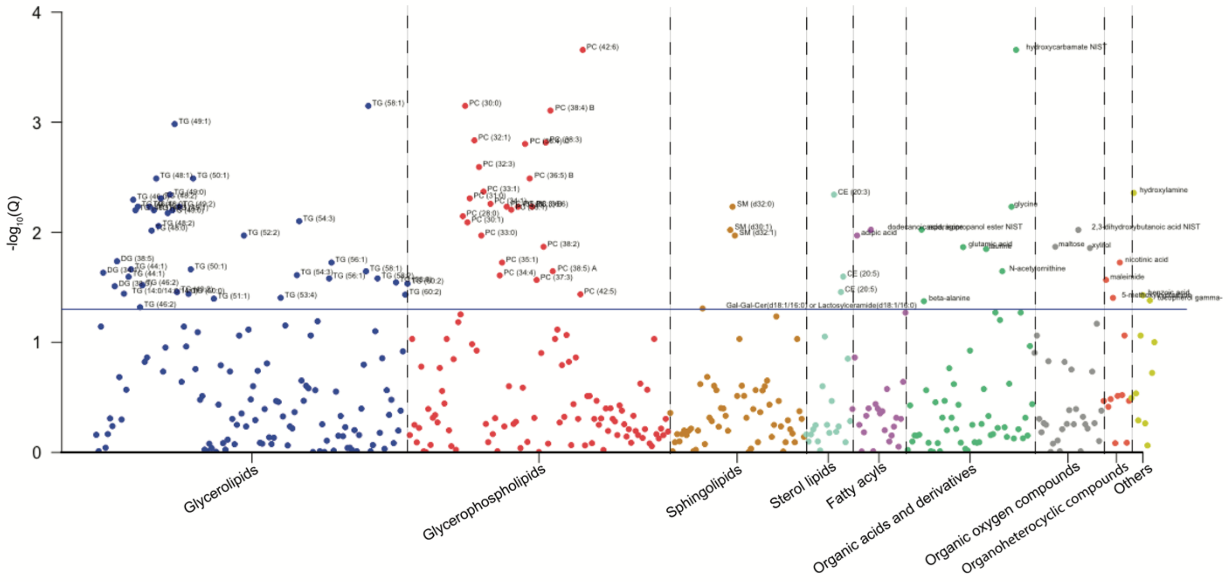

Supplement Figure S1. Manhattan plot for the FDR-adjusted associations (-log$_{10}$ Q values) between DASH score and individual fasting serum metabolite according to metabolic super-class. The horizontal line represent the Benjamini-Hochberg adjusted P values (i.e., Q value) of 0.05. The vertical dashed lines separated metabolites by different super classes. “Others” superclass included nucleosides, nucleotides, and analogues, homogeneous non-metal compounds, benzenoids, organic nitrogen compounds, and prenol lipids, which consisted of less than ten metabolites.
